# Dendritic Cells: Critical Regulators of Allergic Asthma

**DOI:** 10.3390/ijms21217930

**Published:** 2020-10-26

**Authors:** Ioannis Morianos, Maria Semitekolou

**Affiliations:** Cellular Immunology Laboratory, Center for Basic Research, Biomedical Research Foundation of the Academy of Athens, 11525 Athens, Greece; jmor@bioacademy.gr

**Keywords:** allergic asthma, dendritic cells, pulmonary tolerance, cytokines, activin-A

## Abstract

Allergic asthma is a chronic inflammatory disease of the airways characterized by airway hyperresponsiveness (AHR), chronic airway inflammation, and excessive T helper (Th) type 2 immune responses against harmless airborne allergens. Dendritic cells (DCs) represent the most potent antigen-presenting cells of the immune system that act as a bridge between innate and adaptive immunity. Pertinent to allergic asthma, distinct DC subsets are known to play a central role in initiating and maintaining allergen driven Th2 immune responses in the airways. Nevertheless, seminal studies have demonstrated that DCs can also restrain excessive asthmatic responses and thus contribute to the resolution of allergic airway inflammation and the maintenance of pulmonary tolerance. Notably, the transfer of tolerogenic DCs in vivo suppresses Th2 allergic responses and protects or even reverses established allergic airway inflammation. Thus, the identification of novel DC subsets that possess immunoregulatory properties and can efficiently control aberrant asthmatic responses is critical for the re-establishment of tolerance and the amelioration of the asthmatic disease phenotype.

## 1. Introduction

Dendritic cells (DCs) denote the “professional” antigen presenting cells of our immune system and are responsible for the initiation and propagation of effective innate and subsequent adaptive immune responses [1]. DCs are highly specialized immune cells that constantly recognize and sample foreign antigens/allergens and infectious agents at mucosal sites. Upon antigen uptake, DCs migrate to secondary lymphoid tissues and efficiently activate naive CD4^+^ T cells [2]. Depending on the DC subset involved, the cytokine micromilieu and the nature of the activation signal, DCs resourcefully prime CD4^+^ T helper (Th) cells which subsequently differentiate in several distinct subsets, such as Th1, Th2, Th9, and Th17 cells [3]. Apart from the efficient activation of effector T cell-mediated immune responses, DCs are also critically involved in the maintenance of immune homeostasis. Immune homeostasis is accomplished through the employment of several tolerogenic mechanisms, such as the induction of T cell anergy, the instruction of regulatory T (Treg) cells, and the production of regulatory cytokines, such as, IL-10, IL-27, and TGF-beta [4]. It becomes evident that DCs represent an attractive cell-target that can be utilized in vaccination settings aimed at boosting inefficient immune responses for the treatment of infectious diseases and cancer [5,6]. Alternatively, DCs could be utilized to diminish aberrant immune responses in the context of chronic inflammation, autoimmunity, and allergy [5,6]. Pertinent to asthma, a growing body of evidence suggests that the therapeutic transfer of DCs with immunoregulatory properties suppresses aberrant Th2 allergic responses and prevents, or even reverses, established allergic airway inflammation in experimental asthma [7,8,9]. In the first part of this review we briefly describe the immunopathophysiology of type II allergic asthma, as well as the role that DCs play in the instruction of allergen-specific Th-cell mediated immune responses. Moreover, we provide an overview of our current understanding regarding DCs that acquire tolerogenic properties, thus representing critical controllers of excessive Th2-driven allergic airway inflammation in experimental asthma.

## 2. DC Subsets and Their Role in the Regulation of Allergic Asthma

### 2.1. Allergic Asthma

Asthma represents one of the most common chronic inflammatory disorders of the conducting airways that affects over 300 million people worldwide [10,11]. Its prevalence has increased considerably in the last decades, especially among children [10,11]. It is characterized by variable airflow limitation secondary to airway narrowing, increased mucus production, wheezing, dyspnea, chest tightness, and coughing [10,11]. Asthma is usually linked to airway hyperresponsiveness (AHR) to otherwise harmless environmental allergens and chronic airway inflammation. Numerous factors, such as allergens, respiratory infections, exercise, particulate matter/air pollutants, and stress account for the discrepancies and severity of asthma symptoms [10,11]. Asthma has been long considered as a heterogeneous chronic lung disease that comprises several groups of patients characterized by varying characteristics or phenotypes [12]. Depending on the type of the immune cell response that is linked to disease pathogenesis, asthma is categorized as: (a) type 2/allergic asthma, characterized predominantly by Th2 cell-mediated inflammation, and (b) non-type 2 asthma associated mainly with Th1 and/or Th17 cell-mediated inflammation [12]. Allergic asthma represents the most common type of this disorder and is induced by sensitization to innocuous environmental allergens [13]. The latter encompass allergens with proteolytic activity, such as those derived from house dust mites (HDM), grass, weed, tree pollens, fungal spores, animal dander, and occupational sensitizers [13]. After the sensitization phase, the main clinical symptoms of allergic asthma usually occur due to subsequent exposures of the individual to the same allergens [13]. In allergic asthma, several cell types, such as epithelial cells, basophils, mast cells, eosinophils, DCs, B cells, Th2 cells, and Th9 cells hold essential roles in the initiation, maintenance, and propagation of type 2 allergic responses [13]. Nevertheless, DCs that are strategically located in the lung mucosa and continuously sample foreign allergens from the environment represent the most critical players for the induction of allergen-specific Th-cell mediated immune responses in asthma.

### 2.2. Innate and Adaptive Immune Responses in Lung Allergic Inflammation

**Figure 1:**
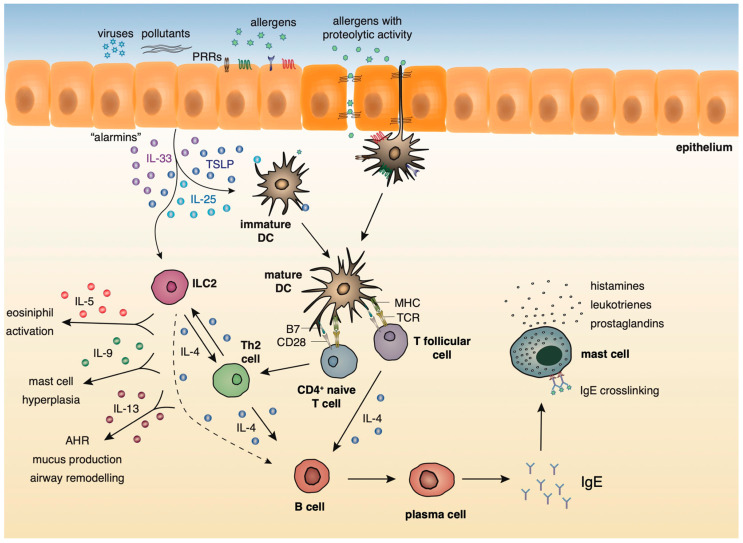
Innate and adaptive type 2 immunity during allergic airway inflammation.

A growing body of evidence underlies the importance of the crosstalk between bronchial epithelial cells and DCs following allergen encounter during the initiation of asthmatic responses [2]. The asthmatic airway epithelium represents a vital supplier of cytokines termed “alarmins”, such as IL-25, IL-33, and thymic stromal lymphopoietin (TSLP) and chemokines, including CCL5, CCL17, CCL11, and CCL22 that trigger Th2 cell polarization upon exposure to allergens, pollutants, and other pathogenic components [14,15,16,17,18,19]. After encountering airborne allergens in the airways, DCs process them into small peptides, generate MHC-peptide complexes and traffic through the lymphatics to the mediastinal lymph nodes (MLNs) where they present allergen components to naive CD4^+^ Th cells [20]. Recent studies have highlighted a critical role for group 2 innate lymphoid cells (ILC2s) in type 2 immunity, as well as in asthma immunopathogenesis [21,22,23,24,25]. ILC2s are activated in response to TSLP, IL-25, and IL-33 signaling, and produce type 2 cytokines (IL-4, IL-5, IL-9, IL-13) [21,22,23,24,25] and prostaglandin [26], further enhancing Th2-driven allergic responses in the airways. Allergens that possess proteolytic activity, such as those derived from HDM, pollen grains, cockroach, and animal and fungal allergens, stimulate protease activated receptors expressed on the surface of DCs and disrupt epithelial tight junctions instigating inflammatory responses [27,28]. Additionally, environmental stimuli, such as respiratory viruses and air pollutants are also capable of disrupting tight junctions and impairing barrier function [29,30,31,32,33]. Furthermore, several allergens contain microbial components named pathogen-associated molecular patterns (PAMPs) that interact with pathogen recognition receptors (PRRs), including Toll-like receptors (TLRs), NOD-like receptors (NLRs), and C-type lectin receptors (CLRs) on DCs and airway epithelial cells, and serve as “danger signals” for the host immune response [34,35]. The interaction of allergen loaded DCs with naive Th cells leads to the differentiation of the latter into Th1, Th2, Th9, or Th17 cells, depending on the type and dose of allergen, as well as, the cytokine micromilieu [36]. In the presence of type 2 cytokines, allergen specific Th2 or T follicular helper cells (TFH) are generated. Th2 and ILC2 cells migrate towards the site of inflammation, where upon allergen challenge, produce type 2 cytokines (IL-5, IL-9, IL-13, etc.). IL-5 and IL-9 are critical for promoting tissue eosinophilia and mast cell hyperplasia, whereas IL- 13 stimulates mucus production by goblet cells and AHR [37]. Seminal studies by Halim and colleagues revealed that IL-13, secreted by ILC2s, stimulates CD11b^+^CD103^−^ lung DCs to produce the chemokine CCL17, which in turn enhances the recruitment of CCR4^+^ memory Th2 cells. Moreover, the depletion of ILC2s in papain-sensitized mice during re-challenge resulted in significantly diminished numbers of IL-4-producing memory Th2 cells in the airways [38]. TFH cells represent a specialized T cell subset that upon interaction with allergen- specific B cells induces the production of IgE antibodies. The crosslinking of these allergen-specific IgE antibodies to the high-affinity FcεR on the surface of mast cells leads to the activation of the latter and the release of several inflammatory mediators, such as, histamine, prostaglandins, and leukotrienes [37] (Figure 1).

During the initiation of asthmatic responses, a plethora of cytokines and chemokines produced by the airway epithelium stimulates immature DCs that upon allergen encounter traffic to the MLNs and stimulate naive CD4+ Th cells. ILC2s, upon activation by the “alarmins”, produce type 2 cytokines further propagating allergic responses. In the presence of type 2 cytokines, allergen specific Th2 or TFH are generated. Th2 cells migrate towards the site of inflammation, where upon allergen challenge, produce mainly, IL-4, IL-5, IL-9, and IL-13. IL-5 and IL-9 are critical for promoting tissue eosinophilia and mast cell hyperplasia, whereas IL- 13 stimulates mucus production by goblet cells and AHR. TFH cells produce IL-4 and, upon interaction with allergen-specific B cells, induce the production of IgE antibodies. The crosslinking of these allergen-specific IgE antibodies to the high-affinity FcεR on the surface of mast cells leads to the activation of the latter and the release of several inflammatory mediators, such as, histamines, prostaglandins, and leukotrienes.

### 2.3. Localization and Phenotype of DC Subsets in the Lung

**Figure 2:**
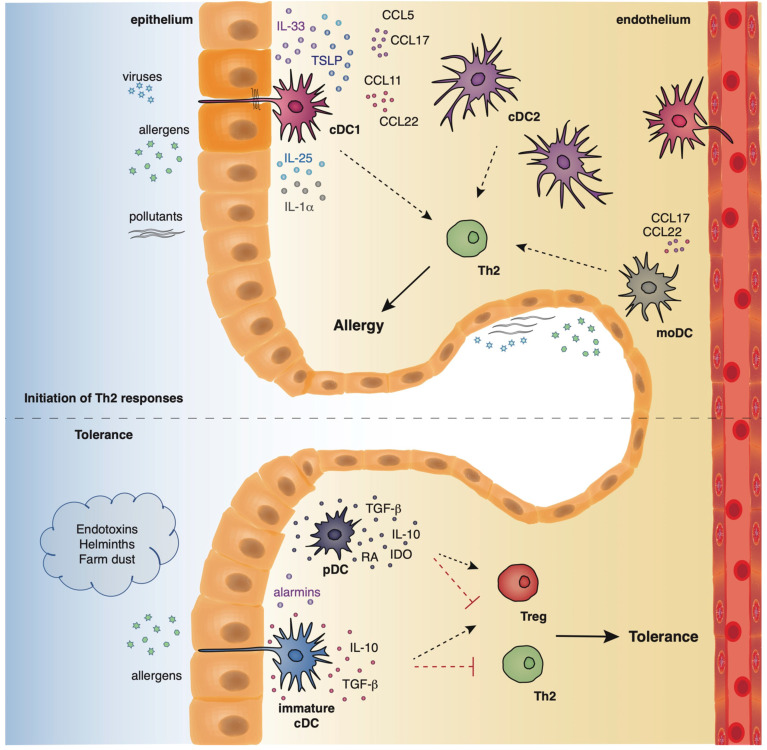
The location and function of different DC subsets in the airways.

Dendritic cells originate from hematopoietic stem cells in the bone marrow from where they start to traffic in order to populate the lymphatic tissues and other organs [39,40]. The divergence in the expression profile of several surface markers, as well as the functional properties of DCs has allowed researchers to classify and characterize different DC subsets that play essential roles in preserving immune homeostasis in the lungs, as well as promoting Th2 immune responses in allergic asthma. Under steady state conditions, mouse pulmonary DCs represent a heterogeneous cell population that mainly comprises of two types of conventional DCs (cDCs), the cDCs type 1 (cDCs1) and type 2 (cDCs2), and also a very small percentage of plasmacytoid DCs (pDCs) [39,40]. Mouse cDCs which are characterized by high expression of the CD11c surface marker are found throughout the lungs, including the large conducting airways, lung parenchyma, alveolar compartment, pleura, and perivascular space. The integrin CD103 is a specific marker for the cDCs1 subset whereas cDCs2 express the cell surface marker, CD11b [41,42,43]. On the contrary, mouse pDCs express low levels of CD11c and are characterized by the expression of other characteristic surface markers, such as, CD45R (B220) and CD317 (PDCA1) [44]. pDCs reside predominantly in the conducting airways, although in a lower density, and have also been described to be present in the lung parenchyma [45]. Under inflammatory conditions, such as, exposure to allergens, pathogens, or pollutants, inflammatory monocyte-derived DCs (mo-DCs) are also recruited to the conducting airways and lung parenchyma [46] (Figure 2). Inflammatory DCs which can be either CD11b^+^ or CD103^+^ originate from Ly6C^+^ blood monocytes under the influence of a granulocyte macrophage colony stimulating factor [40,47].

Pertinent to humans, DCs are generally categorized in analogous subgroups to their murine counterparts. Three pulmonary DCs subsets have been described in humans, two of myeloid origin (mDcs) and one population of pDCs. The mDC1 subset expresses BDCA1 (CD1c), whereas the mDC2 subset expresses BDCA3 (CD141). Human pDCs are mainly characterized by the expression of BDCA2 (CD123) [39,40]. It has been shown that BDCA1^+^ cDCs are increased in the airway epithelium of asthmatics with a high Th2 phenotype, whereas this is not observed in patients with a Th2 low profile [48]. Notably, using unbiased single-cell RNA sequencing, Villani and colleagues provided a revised taxonomy pertinent to human blood DCs. They identified 6 DC populations (DC1-DC6): DC1 cluster is characterized as CD141/BDCA-3^+^CLEC9A^+^, DC2 and DC3 clusters represent subdivisions of CD1c/BDCA-1^+^ cDCs, DC4 cluster is described as CD1c^-^CD141^-^CD11c^+^CD16^+^, DC5 is a cluster defined by the unique expression of the surface markers AXL and SIGLEC6, and DC6 is a cluster that corresponds to the interferon-producing CD123^+^CD303/BDCA-2^+^ pDCs [49,50]. Nevertheless, due to the lack of adequate lung material containing epithelium and interstitium from both healthy controls and asthmatics, the localization of human DC subsets in the asthmatic lung, during steady state and under inflammatory conditions, remains ill defined.

### 2.4. The Role of Murine DCs in the Induction of Allergic Responses in Asthma

Several years ago, van Rijt and colleagues demonstrated that depletion of CD11C^+^ DCs from the airways of CD11c-diphtheria toxin receptor (DTR) transgenic mice, upon the intratracheal injection of DT, during ovalbumin (Ova) aerosol challenge abolished the clinical features of the disease [51]. These included eosinophilic inflammation, goblet cell hyperplasia, and bronchial hyperreactivity, underlying the pivotal role that DCs play in the regulation of the asthmatic response [51]. Both CD103^+^ cDC1s and CD11b^+^ cDC2s, which represent the two major lung DC subsets, have been recognized as critical regulators of allergen-driven immune responses [52,53]. Although CD103^+^ cDC1s are considered to have an inferior capacity to cross-present viral antigens to CD8^+^ T cells [54], it has recently been demonstrated that in response to house dust mite (HDM) or cockroach antigen, this subset of DCs was able to efficiently uptake the allergen and mount an effective Th2 immune response [41,55]. Importantly, mice lacking CD103 exhibited attenuated allergic airway inflammation upon Ova-LPS or HDM inhalation, suggesting that CD103^+^ cDC1s are essential in priming Th2 responses to inhaled allergens [55]. In support to the previous study, another group demonstrated that in an Ova-induced mouse model of experimental allergic airway disease, CD103-knockout BALB/c mice showed reduced airway hyperresponsiveness and eosinophil recruitment to the airways after aerosol challenge compared to wild-type mice [56]. Pertinent to CD11b^+^ cDC2s, this subset is also efficient in allergen uptake and transport to the airways and critical for the induction of Th2-cell differentiation in allergen-exposed lungs [57,58,59]. Several studies have demonstrated that lung CD11b^+^ cDC2s, are necessary for the initiation and generation of effective Th2-mediated adaptive immune responses to inhaled HDM [53,60,61]. It has been also shown that upon a single high dose of HDM, within 48 h, mo-DCs reach the lungs [60]. Although, mo-DCs have the ability to uptake antigen, their capacity to instruct Th-cell proliferation is inferior to cDC2s. Mo-DCs produce substantial amounts of cytokines and chemokines that are critical for the recruitment and activation of Th2 cells upon HDM exposure [60]. In accordance with the aforementioned study, CD11c^+^CD11b^+^ cells isolated from mice following Ova-specific Th2 cell transfer and allergen challenge, produced more CCL17 and CCL22 compared to CD11c^+^/CD11b^−^ cells from the same mice underlying the importance of mo-DCs in recruiting other effector subsets during the effector phase of experimental asthma [61]. Importantly, the depletion of CD11b^+^ cells before Th2 cell transfer and Ova challenge resulted in reduced numbers of Th2 cells and eosinophils in the airways of mice, pointing to the significant role that CD11b^+^ cells play in the induction of allergic airway inflammation [62]. Compared to the other DC subsets, pDCs have a limited capacity to uptake, process, and present antigens [62,63] and due to their suppressive functions in asthma, will be discussed in more detail later in this review during the resolution of allergic airway inflammation. Nevertheless, a study by Lombardi and colleagues showed that CD8α^−^β^−^ pDCs conferred antigenic sensitization in vivo in a model of Ova-induced allergic airway inflammation [64]. In brief, adoptive transfer of Ova-pulsed CD8α^−^β^−^ pDCs into naive mice followed by allergen challenge resulted in enhanced AHR, airway inflammation and mucus production compared to mice that received CD8α^+^β^−^ or CD8α^+^β^+^ Ova-pulsed pDCs [64].

### 2.5. The Role of Human DC Subsets in Shaping Asthmatic Responses

Patients with allergic asthma display heightened numbers of mDC1s and mDC2s in the peripheral blood (PB), induced sputum and bronchoalveolar lavage (BAL) upon allergen inhalation [65,66,67,68]. Nevertheless, upon allergen inhalation only the percentages of cDC2s are increased in bronchial tissues [68]. PB CD1c^+^ mDC2s loaded with *Dermatophagoides pteronyssinus* antigen P1 (Der p1) allergen-IgE immune complexes induced the release of IL-4 and concomitantly lowered IFN-γ-expression. As a result, the use of these DCs in in vitro co-cultures with naive T cells promoted Th2 cell differentiation [69]. Moreover, the co-culture of Der p1-loaded CD1c^+^ mDC2s from asthmatics with allogeneic CD4^+^ T cells from non-asthmatic, non-allergic donors promoted the differentiation of the latter towards a Th2 phenotype [70]. Furthermore, when TSLP-pulsed mDC2s from the same donors were used in the co-cultures, they drove the in vitro development of both Th2 and Th9 cells [70].

Pertinent to the numbers of pDCs in the PB of patients with asthma, there are contradictory results demonstrating that this subset is either increased or decreased [71,72]. Nevertheless, upon allergen challenge the numbers of pDCs are increased in the BAL and sputum of asthmatics [66,73]. During infancy, numbers of circulating pDCs are inversely correlated with symptoms of lower respiratory tract infections, wheezing and asthma diagnosis by 5 years of age [74]. Moreover, co-culture of Derp1–pulsed mo-DCs from HDM-sensitive patients with autologous CD4^+^ T cells resulted in the secretion of large amounts of IL-4 compared to CD4^+^ T cells stimulated with unpulsed DCs. These findings underlie the capacity of mo-DCs to orientate CD4^+^ T cells towards a Th2 response [75]. Importantly, IL-4 production by CD4^+^ T cells from allergic patients was dependent on CD86, as the administration of a blocking antibody against CD86 reduced the secretion of IL-4 [75].

### 2.6. The Role of Tolerogenic DCs in the Resolution of Allergic Airway Inflammation in the Airways

Under physiological conditions, in the absence of a “danger signal”, such as proinflammatory cytokines and microbial products, harmless environmental allergens cannot fully activate lung-derived cDCs or even recruit in the lungs novel inflammatory DCs subsets [76,77]. As a consequence, pulmonary tolerance is achieved as DCs remain in an immature state and are incapable of inducing effective T-cell responses [76,77]. Several studies have shown that DCs not only promote but can also restrain Th2-driven allergic responses through instructing the generation of Treg cells and/or secreting cytokines with immunosuppressive properties, such as IL-10 and TGF-β, suggesting that DCs have acquired tolerogenic/immunosuppressive properties [78,79]. It has been demonstrated that CD103^+^ DCs from OVA-tolerized mice efficiently induced the de novo generation of CD4^+^ Foxp3^+^ Treg cells, a process mediated by retinaldehyde dehydrogenase and peroxisome proliferator-activated receptor γ [78,79]. Importantly, OVA-tolerized Batf3^−/−^ mice that lack CD103^+^ DCs showed increased signs of allergic airway inflammation compared to OVA-tolerized WT mice, underlying the importance of CD103^+^ DCs in the maintenance of pulmonary tolerance [78]. In line with the aforementioned studies, CD103^-/-^ mice sensitized and challenged with OVA or HDM exhibited increased eosinophilic infiltration, severe tissue inflammation, and heightened IL-5 and IFN-γ secretion from lung cells compared to WT mice, strengthening the notion that CD103 DCs display an important role in the control of airway inflammation in asthma [79,80]. Notably, using a HDM-induced chronic asthma model, the authors showed that HDM-immunized Batf3^–/–^ mice developed increased eosinophil and neutrophil influx, enhanced airway inflammation, goblet cell metaplasia, mucus production, and airway collagen deposition compared to WT mice [80]. It has been also demonstrated that in OVA-sensitized and challenged mice, administration of *H. pylori* reduces AHR, bronchoalveolar eosinophilia, pulmonary inflammation, and Th2 cytokine production. Notably, CD103^+^ DCs are required for *H. pylori*-induced protection against allergic airway inflammation as BATF3^−/−^ mice were significantly less protected than WT mice against allergen-induced asthma upon infection with *H. pylori* [81].

Several studies have shown that bone marrow-derived DCs (BM-DCs) exposed in vitro to IL-10 and/or TGF-β1 decreased AHR, airway eosinophilia, Th2 cytokine release, as well as IgE levels upon transfer in models of Ova-induced allergic airway inflammation [82,83,84]. Similar results were obtained following the administration of IL-10-stimulated, HDM-loaded BM-DCs to HDM-sensitized and challenged mice [85]. Importantly, mice administered with IL-10-treated BM-DCs in a tolerance-inducing protocol, exhibited reduced allergic disease manifestations and Th2 responses that remained suppressed for up to 8 months [86]. Moreover, the adoptive transfer of dexamethasone and LPS-treated BM-DCs prior to sensitization of mice with *Blomia tropicalis mite* (Bte) extract and aluminium hydroxide resulted in reduced numbers of inflammatory cells in the BAL and lungs, mucus deposition and serum levels of anti-BtE IgE antibodies compared to PBS-injected controls [87]. In addition, the intratracheal or intravenous administration of OVA-pulsed, BM-derived DCs treated ex vivo with heme oxygenase-1 to BALB/c mice before allergen challenge led to decreased lung inflammation concomitant with the generation CD4^+^ Foxp3^+^ T reg cells in the BAL compared to mice treated with vehicle [88]. Likewise, adoptive transfer of splenic DCs pulsed in vitro with OVA/flagellin-B (FlaB) to OVA-sensitized and challenged mice ameliorated all the cardinal features of allergic airway inflammation, dampened OVA-specific IgE serum levels, as well as Th2 cytokine release from MLN cell culture supernatants upon ex vivo allergen exposure [89]. The authors observed the same results using also an HDM-induced experimental model of allergic airway inflammation [89]. Of clinical importance, FlaB-treated DCs isolated from the PB of patients with HDM-sensitive asthma were able to drive the generation of CD4^+^Foxp3^+^ Treg cells upon co-culture with autologous CD4^+^ T cells in the presence of HDM extracts, a process mediated by IL-10 release in cell culture supernatants [89].

Pertinent to the role of pDCs in the regulation of asthmatic responses, several studies have demonstrated that pDCs confer protection against allergic airway inflammation through at least the instruction of T reg cells [90,91]. Notably, the depletion of pDC before the sensitization phase with Ova resulted in increased lung inflammation and Th2 cell cytokine production from MLNs compared to WT mice with sufficient numbers of pDCs, revealing that pDCs restrain sensitization to otherwise harmless tolerogenic protein antigens [92]. In line with the above, the in vivo depletion of pDCs before Ova challenge resulted in heightened numbers of eosinophils and lymphocytes in the airways of mice [93]. Notably, the in vivo administration of OVA-pulsed pDCs before allergen challenge resulted in a strong decrease in the numbers of BAL fluid eosinophils and lymphocytes, underlying the regulatory role of pDCs in allergic airway inflammation [93]. Moreover, the in vivo transfer of Ova-pulsed CD8α^+^β^−^ or CD8α^+^β^+^ pDCs before allergen sensitization and challenge prevented the induction of AHR and lung inflammation and decreased Th2 cell cytokine production from MLNs and OVA-specific IgE serum levels [64]. Nevertheless, another study showed that in an acute model of allergic airway inflammation, pDC depletion after Ova challenge reduced AHR, the numbers of eosinophils and T cells in the BAL fluid, as well as peribronchial and perivascular inflammation and OVA-specific Th2 responses in MLNs, suggesting that pDCs promote allergic inflammatory responses in the airways once disease has been established [94].

A plethora of studies have discovered several factors, such as the type of antigen sampled, the route of administration, the existence of microbial patterns or endogenous danger signals in the microenvironment and the genetic background of the host that dictate whether DCs will mount a robust Th2-driven allergic response or acquire tolerogenic properties [95,96]. In the case of experimental allergens, such as ova which do not possess intrinsic activating properties like allergens with proteolytic activity, additional signals from contaminating molecules or environmental exposures (viruses, air pollutants or cigarette smoke) are needed to initiate DC activation [97,98]. For example, in an ova-induced mouse of allergic airway inflammation, when ova is administered in conjunction with the adjuvant aluminium hydroxide (alum) mice develop all the clinical features of experimental asthma whereas when ova is administered in the absence of an adjuvant, mice become tolerant to this antigen and the development of allergic inflammation in the airways is sustained [99,100]. Importantly, in human subjects with genetic susceptibility, the exposure of DCs to allergens, in a microenvironment comprised of low amounts of PAMPs and DAMPs, leads to enhanced priming of DCs and subsequent allergic responsiveness [101]. In contrast, when DCs are exposed to abundant PAMP- and DAMP-mediated signals (farm life, traditional lifestyles, etc.) allergenic tolerance is established [102]. Environmental changes that occur in the microenvironment are sensed by the epithelium and DCs at mucosal sites, such as the gut, the skin and the lungs and subsequently modify the context of allergen recognition and either protect from or initiate type 2-driven immune responses [103].

Recently, Haspeslagh and colleagues proposed a model of airway tolerance that underlies the factors that dictate whether a DC will promote type 2 immunity or become tolerogenic. In brief, in the absence of regulatory factors, the epithelium secretes inflammatory mediators, such as IL-1α, IL-25, IL-33, CCL20, upon the binding of allergens on PRRs. These inflammatory mediators license antigen loaded IRF4^+^ CD11b^+^ conventional DCs to polarize naïve T cells towards Th2 cells in the MLNs. Neonatal and germ-free mice are especially susceptible to develop such type 2 responses. Respiratory syncytial virus infections and cigarette smoke upregulate IL-33 secretion and thus may promote this pathway. On the contrary, exposure to endotoxins, farm dust, or microbial colonization reduces the epithelial response by increasing the expression of negative regulators. One such example are helminth-derived products that diminish the production of IL-33 from the epithelium. DCs devoid of epithelial activation signals are unable to initiate T cell activation. In addition, DCs from microbe-exposed animals confer protection against disease manifestations by inducing the generation of regulatory T cells (Tregs) [103] (Figure 2).

Allergens with proteolytic activity, air pollutants, and viruses (asthma risk factors) stimulate the epithelium to produce alarmins and inflammatory factors that activate cDCs and moDCs which in turn initiate and propagate Th2-driven allergic responses. On the contrary, exposure to endotoxins, helminths, and farm dust reduces the epithelial responses, favoring the maintenance of cDCs in an immature state. Along with immature cDCs, pDCs produce immunosuppressive factors, facilitating the establishment of pulmonary tolerance.

### 2.7. A Novel Subset of Tolerogenic DCs Confers Protection against Experimental Asthma

Although a growing body of evidence suggests that therapeutic transfer of DCs with immunoregulatory properties inhibits pathogenic Th2 effector responses and prevents or even reverses established airway inflammation in experimental asthma [7,8], the tolerogenic responses mediated by DCs are often impaired or overridden in human asthma [104]. Therefore, the identification of novel mechanisms that induce and/or restore the immunoregulatory properties of DCs in the airways is critical for the design of novel therapeutic approaches for the treatment of human asthma. Previous studies by our group demonstrated that the pleiotropic cytokine activin-A instructs the generation of Foxp3^-^, IL-10-producing Treg cells (act-A-iTreg cells) that exhibit robust suppressive functions towards allergen-induced naive and Th2 effector responses. Importantly, the therapeutic administration of act-A-iTreg cells in an Ova-induced allergic airway inflammation mouse model conferred significant protection against asthma manifestations, associated with markedly decreased frequencies of activated CD11c^+^MHCII^high^ cDCs in the lung-draining MLNs [105]. Additionally, act-A-iTreg cells upon in vivo interaction with DCs endowed the latter with enhanced tolerogenic functions (act-A-iTreg-modified DCs).

**Figure 3:**
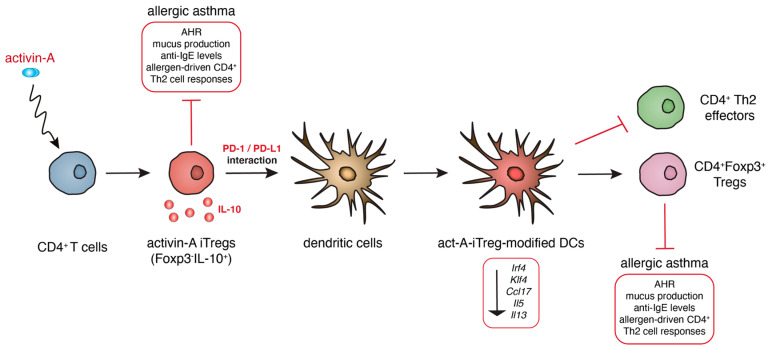
Act-A-iTreg-modified DCs control allergic asthma through the instruction of CD4^+^Foxp3^+^ regulatory T cells.

Notably, act-A-iTreg-modified DCs exhibited a significantly impaired capacity to sample allergen and traffic to the MLNs, accompanied by a markedly decreased ability to prime Th2 responses in vivo [105]. Despite extensive research, the precise transcriptional programs that underlie the tolerogenic properties of DCs in the context of asthmatic responses remain largely unidentified. Interestingly, our transcriptional analyses revealed that act-A-iTreg-modified DCs displayed a prominent reduction in a plethora of genes associated with Th2 cell-priming potential, including the transcription factors, IRF4 and KLF4, Th2 cytokines, and chemokines and their signaling pathways. Of clinical relevance, vaccination of allergic mice with act-A-iTreg-modified DCs conferred protection against experimental asthma through the generation of Foxp3^+^ Treg cells in a feed-forward immunoregulatory circuit. Mechanistically, our studies revealed that PD-1/PD-L1 interactions are critical for the generation of DCs with immunoregulatory properties by act-A-iTreg cells [105]. Collectively, our findings unraveled a crucial role for act-A-iTreg cells in potentiating the generation of tolerogenic DCs that can be harnessed for the control of aberrant Th2-mediated allergic airway inflammation and linked asthmatic disease (Figure 3).

The cytokine activin-A induces the generation of IL-10-producing Treg cells (act-A-iTreg cells) that upon therapeutic administration confer significant protection against asthma manifestations. Notably, act-A-iTreg cells upon in vivo interaction with DCs endow the latter with enhanced tolerogenic functions (act-A-iTreg-modified DCs), a process that is dependent on PD-1/PD-L1 interactions. Act-A-iTreg-modified DCs display a prominent reduction in a plethora of genes associated with Th2 cell-priming potential. Of clinical relevance, vaccination of allergic mice with act-A-iTreg-modified DCs protected against experimental asthma through the generation of Foxp3^+^ Treg cells.

## 3. Conclusions

Emerging evidence underlies the vital role that DCs play in fine tuning the balance between protective immunity and pulmonary tolerance in the airways in the context of allergic asthma. The functions of different DC subsets during the initiation and propagation of allergic asthma are very complex and highly dependent on both the nature and the levels of inhaled allergen, route of administration, composition of the local microenvironment, localization of DCs in the airways, as well as the phase of the immune response. As mentioned above, several studies have revealed that cDC1s, cDC2s, and mo-DCs are critical in orchestrating inflammatory responses in the airways, whereas CD103^+^ cDC1s and pDCs mainly contribute to the resolution of inflammation in the airways through the release of immunoregulatory cytokines, such as IL-10 and TGF-β, and the instruction of Treg cells. Although vaccination strategies involving the therapeutic transfer of tolerogenic immune cells are increasingly being utilized to limit detrimental self-reactive responses in autoimmune diseases, their use in the control of allergic responses has not been exploited.

Since DCs represent major drivers of immune polarization, they could be used as potential therapeutic targets in several diseases, including allergic asthma. Currently, allergen-specific immunotherapy (AIT), involving exposure of individuals to escalating concentrations of an allergen, represents the only approved therapy for the control of allergic disorders, including asthma (108). The primary objective of AIT is to reestablish peripheral immune tolerance towards allergens, and diminish early- and late-phase allergic reactions through the induction of allergen-specific regulatory cell subsets, the production of suppressor cytokines (e.g., IL-10 and TGF-b) and inhibitory molecules (e.g., PD-1 and CTLA-4) and limitation of IgE production [106]. Towards the same direction, the administration of genetically engineered tolerogenic DCs could induce allergen-specific tolerance, holding the potential to be used as a therapeutic regime in allergic disorders. Indeed, monocyte-derived DCs from atopic individuals, treated ex vivo with the immunosuppressive agent IL-10, were able to suppress allergen-induced T-cell proliferation of autologous naive and memory CD4^+^ T helper cells and type 2 cytokine release [107]. In the context of contact dermatitis, dexamethasone-treated DCs from the peripheral blood of individuals with IgE-mediated latex allergy restrain allergen-specific T cell proliferation and IgE production in vitro and induce the generation of IL-10-producing Tregs [108]. A better understanding of the underlying cellular and molecular pathways involved in the generation of novel DC subsets that possess immunosuppressive properties may facilitate the design of novel DC-based immunotherapies for the re-establishment of tolerance in the airways.

## Abbreviations

AHR	airway hyperresponsiveness
Bte	Blomia tropicalis mite
cDCs	conventional DCs
cDCs1	cDCs type 1
cDCs2	cDCs type 2
DCs	dendritic cells
Der p1	Dermatophagoides pteronyssinus antigen P1
FlaB	flagellin-B
HDM	house dust mite
MLNs	mediastinal lymph nodes
mo-DCs	monocyte-derived DCs
mDCs	myeloid DCs
Ova	ovalbumin
PB	peripheral blood
pDCs	plasmacytoid DCs
Th	T helper
TFH	T follicular helper
Treg	Regulatory T cell
TSLP	Thymic stromal lymphopoietin
WT	wild type
